# Endoluminal visualization of distal airway abnormalities within a ground-glass opacity during robotic-assisted bronchoscopy

**DOI:** 10.1093/ajrccm/aamag301

**Published:** 2026-06-22

**Authors:** Edoardo Amante, Alessandra Cancellieri, Filippo Lococo, Qianqian Zhang, Samy Lachkar, Rocco Trisolini

**Affiliations:** Interventional Pulmonology Unit, Fondazione Policlinico Universitario “Agostino Gemelli” IRCCS, Roma, Italy; Pathology Unit, Fondazione Policlinico Universitario “Agostino Gemelli” IRCCS, Roma, Italy; Thoracic Surgery Unit, Fondazione Policlinico Universitario “Agostino Gemelli” IRCCS, Roma, Italy; Pathology Unit, Fondazione Policlinico Universitario “Agostino Gemelli” IRCCS, Roma, Italy; Department of Pulmonology, UNIROUEN, AIMSQuantif, CHU de Rouen, University of Normandie, Inserm CIC-CRB 1404, Rouen, France; Interventional Pulmonology Unit, Fondazione Policlinico Universitario “Agostino Gemelli” IRCCS, Roma, Italy; Department of Cardiovascular and Pulmonary Sciences, Catholic University of the Sacred Hearth, Roma, Italy

A 62-year-old never-smoking woman was referred for robotic-assisted bronchoscopy to evaluate a 2.9-cm pure ground-glass opacity (GGO) in the left upper lobe, incidentally detected on chest radiography and confirmed by high-resolution computed tomography (HRCT). Follow-up HRCT performed 4 months later showed no interval change in lesion size or attenuation.

Robotic-assisted bronchoscopy was performed using the iON platform (Intuitive Surgical). A high-definition, miniaturized 1.3-mm video probe (Iriscope; Lys Medical) was advanced toward the target through its dedicated 2.0-mm outer diameter delivery catheter, which incorporates a port for saline or air instillation to maintain lens visualization. Following navigation to the target segment with the robotic bronchoscope, the probe-catheter assembly was advanced distally toward the target lesion, with positioning within the GGO confirmed using cone-beam CT (Artis Pheno; Siemens) (Figure 1A-D). Endoluminal imaging revealed diffuse abnormal mucosal irregularities of the distal airways within the target region (Figure 1E-F; Video E1 in the online supplement). The probe was withdrawn, and multiple forceps biopsy were obtained through the guide sheath under fluoroscopic guidance. Histopathological examination of bronchoscopic specimens demonstrated adenocarcinoma with predominant lepidic growth. The patient subsequently underwent left upper trisegmentectomy, which confirmed minimally invasive adenocarcinoma, defined by a predominantly lepidic pattern with an invasive component measuring ≤ 5 mm (Figure 2).

**Figure 1:**
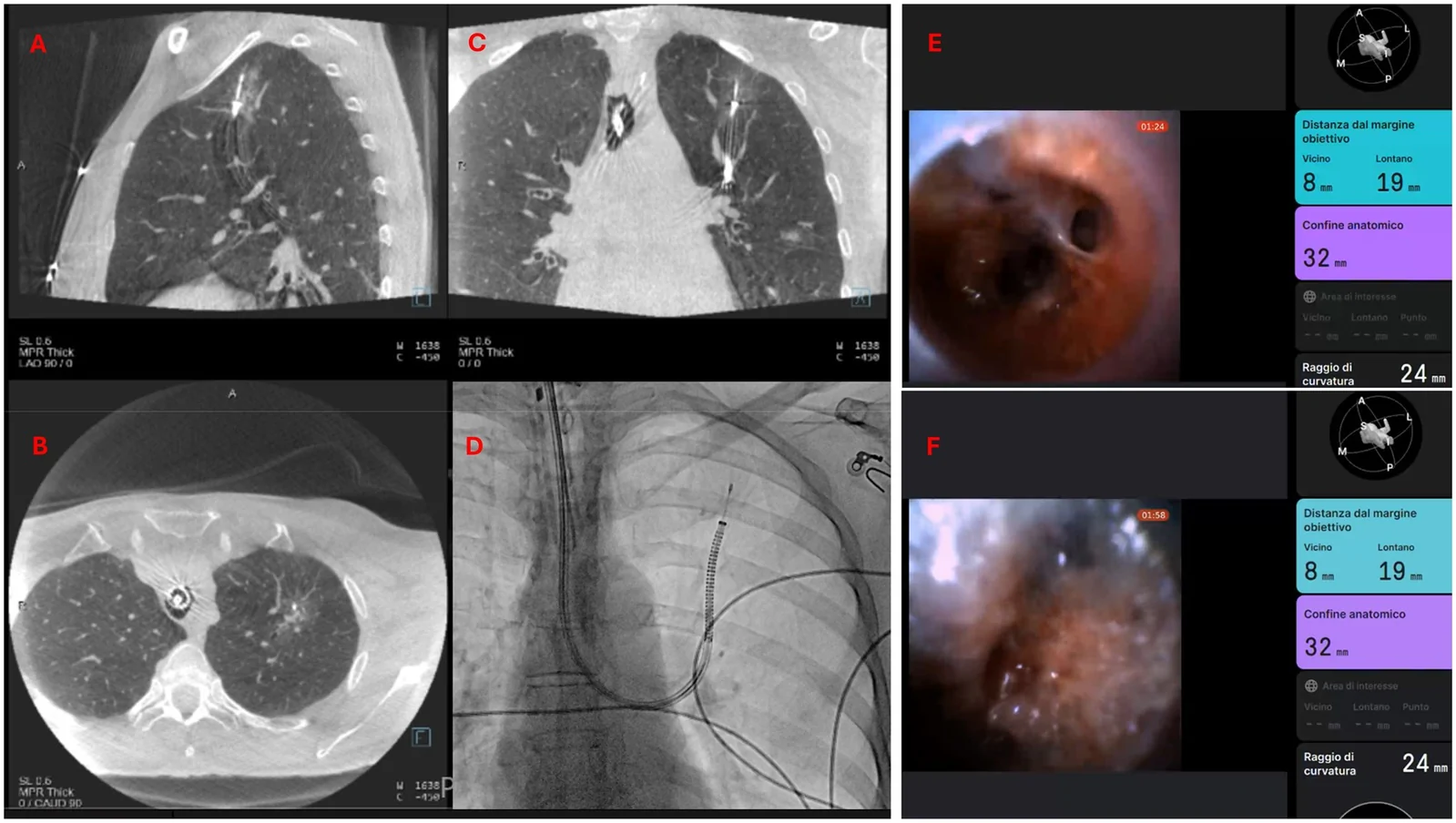
Cone-beam computed tomography images confirming correct positioning of the Iriscope video probe within the ground-glass opacity in sagittal (A), axial (B), and coronal (C-D) planes. High-definition endoluminal images obtained with the Iriscope demonstrate diffuse mucosal ­irregularities of the distal airways within the target region (E-F).

**Figure 2:**
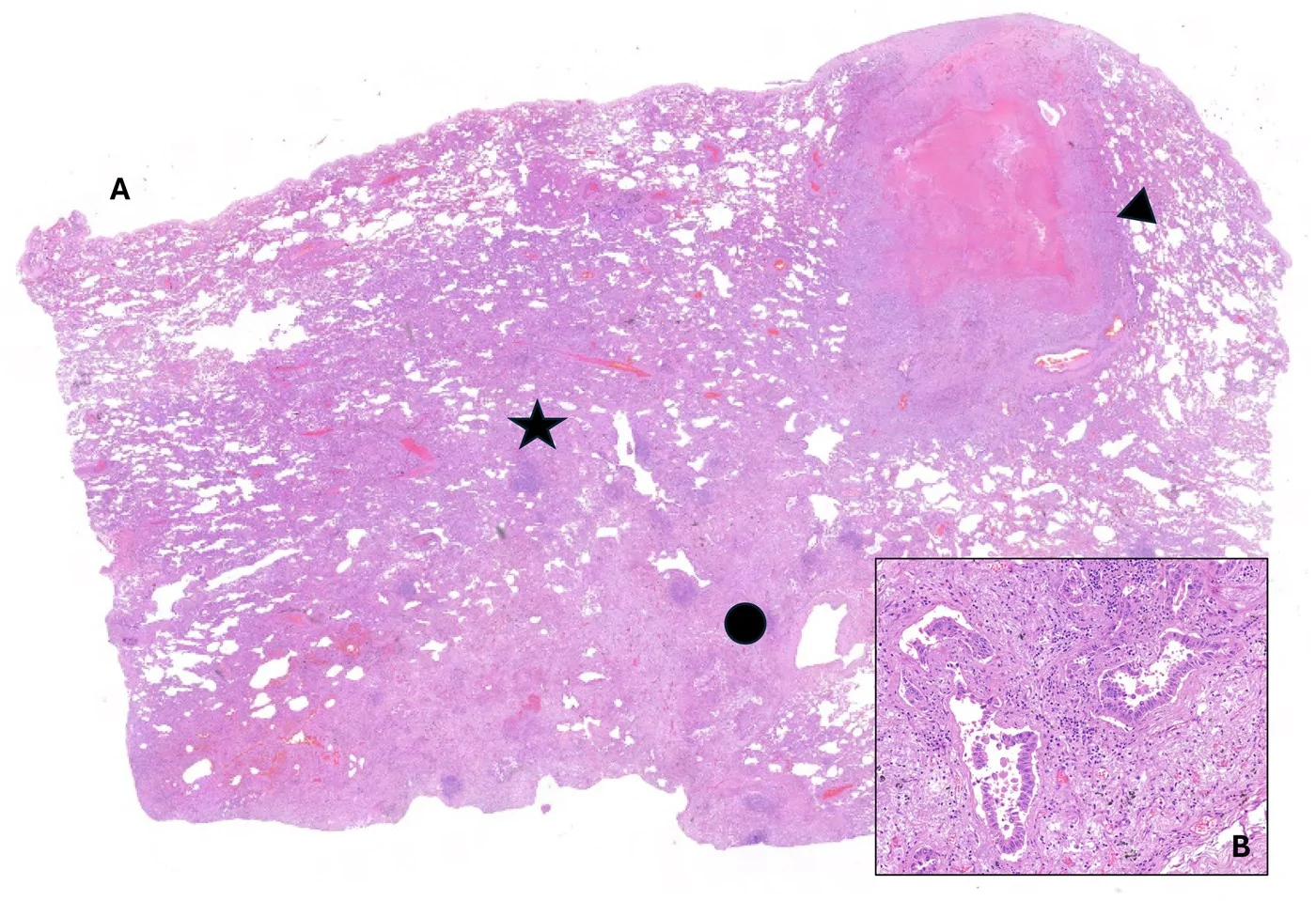
(A) Low-power photomicrograph of the left upper trisegmentectomy specimen showing a minimally invasive adenocarcinoma with a predominant lepidic growth pattern (star) and a focal invasive component (center). A hemorrhagic area with surrounding fibrosis, consistent with the prior biopsy site, is also visible (arrowhead). Hematoxylin and eosin (H&E) stain; original magnification × 4. (B) Higher-power inset showing nonmucinous acinar adenocarcinoma glands infiltrating a sclero-elastotic stroma. H&E stain; original magnification × 80.

This case illustrates the endoscopic evaluation of a large pure GGO and demonstrates that distal airway abnormalities within GGOs can be directly visualized in vivo using high-definition endoscopic video probes.^1^^,^^2^

## Supplementary Material

aamag301_Supplementary_Data

## Data Availability

This article has an online data supplement, which is accessible from this issue’s table of content online at www.atsjournals.org.
