# Supplementary material for: Endoluminal visualization of distal airway abnormalities within a ground-glass opacity during robotic-assisted bronchoscopy
Source: Am J Respir Crit Care Med. 2026 Jun 22;212(10):2634–5. doi: 10.1093/ajrccm/aamag301 (PMC13624804; doi:10.1093/ajrccm/aamag301)
Supplement: Supplementary file 1 [file aamag301_supplementary_data.zip › Legend_to_Supplemental_Video_E1.docx]

Supplemental Video Legend

Endoluminal inspection with the Iriscope showing diffuse abnormal mucosal irregularities of the distal airways within the ground-glass opacity.
